# Corpus-Based Discourse Analysis of a Reddit Community of Users of Crystal Methamphetamine: Mixed Methods Study

**DOI:** 10.2196/48189

**Published:** 2023-09-29

**Authors:** Andrew Lustig, Gavin Brookes

**Affiliations:** 1 Department of Psychiatry Temerty Faculty of Medicine University of Toronto Toronto, ON Canada; 2 Department of Linguistics and English Language Lancaster University Lancaster United Kingdom

**Keywords:** methamphetamine, social media, substance-related disorders, discourse analysis, mental health, mixed methods, corpus analysis, web-based health

## Abstract

**Background:**

Methamphetamine is a highly addictive stimulant that affects the central nervous system. Crystal methamphetamine is a form of the drug resembling glass fragments or shiny bluish-white rocks that can be taken through smoking, swallowing, snorting, or injecting the powder once it has been dissolved in water or alcohol.

**Objective:**

The objective of this study is to examine how identities are socially (discursively) constructed by people who use methamphetamine within a subreddit for people who regularly use crystal meth.

**Methods:**

Using a mixed methods approach, we analyzed 1000 threads (318,422 words) from a subreddit for regular crystal meth users. The qualitative component of the analysis used concordancing and corpus-based discourse analysis to identify discursive themes informed by assemblage theory. The quantitative portion of the analysis used corpus linguistic techniques including keyword analysis to identify words occurring with statistically marked frequency in the corpus and collocation analysis to analyze their discursive context.

**Results:**

Our findings reveal that the subreddit contributors use a rich and varied lexicon to describe crystal meth and other substances, ranging from a neuroscientific register (eg, methamphetamine and dopamine) to informal vernacular (eg, meth, dope, and fent) and commercial appellations (eg, Adderall and Seroquel). They also use linguistic resources to construct symbolic boundaries between different types of methamphetamine users, differentiating between the esteemed category of “functional addicts” and relegating others to the stigmatized category of “tweakers.” In addition, contributors contest the dominant view that methamphetamine use inevitably leads to psychosis, arguing instead for a more nuanced understanding that considers the interplay of factors such as sleep deprivation, poor nutrition, and neglected hygiene.

**Conclusions:**

The subreddit contributors’ discourse offers a “set and setting” perspective, which provides a fresh viewpoint on drug-induced psychosis and can guide future harm reduction strategies and research. In contrast to this view, many previous studies overlook the real-world complexities of methamphetamine use, perhaps due to the use of controlled experimental settings. Actual drug use, intoxication, and addiction are complex, multifaceted, and elusive phenomena that defy straightforward characterization.

## Introduction

Methamphetamine is a highly addictive stimulant that affects the central nervous system. Crystal methamphetamine (or “crystal meth”) is a form of the drug resembling glass fragments or shiny bluish-white rocks that can be taken through smoking, swallowing (pill), snorting, or injecting the powder once it has been dissolved in water or alcohol [1]. This study examines the social construction of identity among people who use methamphetamine. Using a corpus-based approach to discourse analysis (introduced in the Methods section), we interrogate a data set (or “corpus”) comprising 500 threads from a subreddit for people who regularly use crystal meth. More specifically, our analysis takes an inductive approach that seeks to identify discursive themes within the threads, using exploratory methods within the corpus linguistics toolkit (introduced in more detail in the Methods section). Our interpretation of observed themes is informed by assemblage theory (introduced in the next section).

Methamphetamine has received extensive media, policy, and research attention that has emphasized its perceived association with violence, physical deterioration, and mental health problems [2]. Government agencies, scientists, and others have characterized methamphetamine as dangerous [3]. Chronic methamphetamine use has been implicated in memory loss [4], psychosis [5], cognitive deficits [6], mood disturbances [7], and dental problems [8]. Both the scholarly literature and the popular press [9] have positioned methamphetamine use and its effects as a public health crisis. Because its use is highly stigmatized in some parts of society [10], methamphetamine use is often carried out in secret or only with sympathetic fellow users to avoid the judgment of those who do not endorse such practices [2].

Social media affords an opportunity to openly and honestly discuss a variety of habits, practices, and health conditions, which may be difficult to address offline [11] due to the theorized “online disinhibition effect” [12]. Some social media platforms promote more radically honest disclosure by granting participants anonymity or pseudonymity [13,14], which may facilitate more frank disclosure about practices and attitudes toward drug use. The social media platform Reddit in particular permits the use of characteristic “throwaway” accounts which can be discarded after a single use. This allows users to abandon an account after posting information that they do not want to be associated with their other web-based activity or their offline identity [15]. Barak et al [16] have described how participation in digital support groups allows people with particular experiences to share information, provide and accept emotional support, socialize, and form relationships [16].

In contrast to the treatment of opioid use disorders, where there is a robust evidence base supporting the use of agonist therapy such as methadone and buprenorphine in treatment, a similar approach has not been found to be effective in the treatment of amphetamine-type stimulant use disorders. Recent meta-analyses have demonstrated that agonist therapy—that is, prescribing stimulants for the treatment of amphetamine-type stimulant use disorder—has no use in promoting sustained abstinence in people with methamphetamine use disorder [17]. Such efforts at treatment are based on the presumption that addiction exists independently of efforts to describe it. This medically informed model of methamphetamine addiction views it as a disease that is the result of disordered brain processes. Neurochemical [18], neurophysiological [19], and genetic [20] explanations are invoked in the literature to explain the effects of methamphetamine use, as well as the syndromes of addiction and dependence. These explanations embrace the disease model of addiction but ultimately fail to provide a coherent view of the complex nature of addiction [21].

An assemblage approach to amphetamine use offers some insight into conceptualizing substance use in its full complexity. This approach does not dispute the biological basis of addiction but adds to it by describing the manifold other factors that bear upon addictive behaviors. It conceptualizes addiction as a collection of heterogeneous bodies, objects, and forces that form a complex of addiction. Examples of such factors include the people one associates with while using drugs, the spaces where drugs are consumed, cultural mores regarding drug use, and economic considerations determining the price of drugs at the site of consumption. It conceptualizes drug use and drug addiction as a dynamic collection of heterogeneous elements including bodies, materials, spaces, forces, and signs [22]. Assemblage thinking serves to emphasize the real conditions in which drug problems emerge by way of the entire cast of human and nonhuman, distal, and proximate forces at work in such problems [22].

In this study, we provide what is, to our knowledge, the first examination of how methamphetamine use as a habit and as an addiction is discursively produced, reproduced, and contested on a subreddit specifically for people using methamphetamine. Because of its unstructured nature, this site permits contributors to discuss drug use in its full complexity without biases that may be introduced by structured or semistructured interview guides. In contrast to studies that recruit diagnostically homogenous groups, this subreddit is available to anyone with an internet connection. This admits the possibility of a heterogeneous, messy collection of contributors who relate to methamphetamine use in different ways.

Although much public health research about methamphetamine has focused on its harms, a more recent strand of research advocates a more comprehensive and inclusive approach to scholarship around drug use and addiction, contemplating both its harms and pleasures [23]. This study has the advantage of collecting data from an open forum (ie, a subreddit) where contributors can write about any topic pertaining to methamphetamine use of their choosing, in the process discursively constructing, theoretically, any kind of relationship between themselves and methamphetamine use. In this way, such interactional contexts provide promising sites for adopting a more comprehensive and inclusive approach to understanding this topic and how users relate to this in terms of their “normal life.”

This study represents, to our knowledge, the first digital ethnography of a web-based community of methamphetamine users and provides novel insights into the discursive practices through which this community, and indeed the practice of methamphetamine use are socially constructed. In particular, the analysis reveals a different perspective on drug-induced psychosis compared to conventional scholarly literature, with methamphetamine presented as not necessarily inducing psychosis, when paired with regular sleep, good nutrition, and meaningful activities. We will argue that this perspective challenges the traditional view that directly links drug use to psychosis, highlighting the significance of context, setting, and other behaviors in these experiences. This research thus sheds new light on the discursive dynamics surrounding real-world drug use, hinting at its complexity and multifaceted nature. Following the reporting of the results, we consider how the insights produced through our analysis may guide harm reduction efforts and inform future research.

## Methods

### Overview

We used a corpus-based approach to discourse analysis [24,25]. Corpus linguistics is a methodology and a field of research that uses computational and statistical techniques to examine linguistic patterns in large, digitized bodies of naturally occurring language use [26,27]. The data analyzed in a corpus linguistic study is referred to as the “corpus” (pl. “corpora”). A corpus comprises a large body of naturally occurring language designed to represent a language or specific context of language use. The language or texts in a corpus are typically digitized to enable computational analysis. With computational assistance, those using corpus linguistic methods can identify recurrent patterns of language use in vast corpora quickly and reliably, and also easily perform complex statistical procedures to measure the significance and strength of the patterns observed.

The corpus we constructed for this study—referred to henceforth as the “methamphetamine corpus”—–represents interactions taking place in a subreddit for self-identified users of methamphetamine. We used Python (version 3.0; Python Software Foundation) to extract the 1000 most recent posts and all associated comments made prior to July 6, 2022, the date of data collection. Posts that were posted and subsequently deleted were not available for analysis (n=137). In total, 863 complete posts (370,452 words; see Textbox 1) were analyzed. The subreddit requires posters to solve a CAPTCHA before posting to prove they are human and not a bot.

At the time of sampling, the subreddit had approximately 107,000 members. To ensure that subreddit members’ identities are protected as far as possible, no usernames or references to any other personally identifying information will be reproduced in the data extracts cited in this paper.

The corpus-based approach to discourse analysis used in this study is inductive and draws upon a combination of techniques such as keyword analysis, collocation analysis, and manual examination of concordances. We used Sketch Engine [28] to compile a list of keywords for the methamphetamine corpus. Keywords are essentially words that appear with a higher than expected frequency in the study corpus when compared to a reference corpus. The reference corpus typically represents some “benchmark” of language use against which the language in the study corpus can be compared. In this case, we obtained keywords for the methamphetamine corpus by comparing it against enTenTen20, a 36 billion-word corpus representing the English language used on the internet [29]. We used the simple math method of calculating keyness scores and we included all keywords with a keyness score ≥50 in our analysis. The simple math method of calculating keywords contains a smoothing parameter.



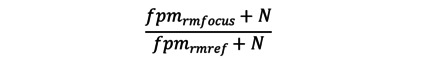



where *fpm_rmfocus_* is the normalized (per million) frequency of the word in the focus corpus; *fpm_rmref_*is the normalized (per million) frequency of the word in the reference corpus, and *N* is a smoothing parameter.

We used *N*=1 as the smoothing parameter, which is the default value in Sketch Engine, for this analysis. As noted, we used keyword analysis inductively—as a “way in” to identify the characteristic language use and various themes and objects of the discourse produced by the subreddit users in their posts. As will be demonstrated in the Results section, the keywords produced through this step, when inspected manually in their contexts of use, could be grouped into a series of thematic and lexical categories, which reflect the kinds of themes and language use that were characteristic of the interactions in the threads in our corpus relative to general English language use on the internet.

We then examined the use of several keywords of interest using collocation analysis. Collocation is the linguistic device, whereby pairs of words (and sometimes wider networks of words) become bearers of meaning through repeated patterns of co-occurrence in natural language [26,27]. Through their collocational relationships, then, a word can develop meanings and exhibit particular “discourse prosodies” or carry particular ideologies [30]. Discourse prosodies broadly refer to the relationships between words and the textual contexts in which they are embedded. Collocation analysis involves identifying patterns of word co-occurrence, which are more frequent in the corpus than would be expected by chance. Collocational pairings were examined within a 5-word window on either side of the search term (or “node”) and ranked using the cubed mutual information statistic. We analyzed collocations using *WordSmith Tools* (Lexical Analysis Software). We also analyzed collocational relationships between words through lexical bundles or n-grams [31]. These are fixed multiword expressions where 2 or more words occur together.

Finally, we followed up our identification of collocational pairings and n-grams with a qualitative discourse analysis, using concordancing, where we examined instances of a particular word or n-gram in context in the study corpus. For this portion of the study, we relied on the critical analysis of the concept of addiction employed by Keane [32]. This approach problematizes the binary opposition of health and disease and recognizes that addiction is not “a universal feature of human existence, but a culturally specific way of understanding, classifying and regulating particular problems of individual conduct.” Following Fraser et al [33], we also draw upon the notion of collateral realities described by John Law. Collateral realities refer to realities that are the product of an ontological politics and assert that realities are done in practice and must be “done and redone again to remain stable.” This approach regards addiction as an assemblage—a heterogeneous collection of objects, forces, affects, and tendencies—that is unstable and characterized by relations of exteriority.

The outputs of the corpus linguistic procedures were initially interpreted by the first author (AL), with the interpretations then being checked for plausibility by the second author (GB; as this was a plausibility check, this did not involve generating a formal intercoder reliability score for thematic interpretation). The discursive themes and wider patterns reported in the next section were agreed upon by both researchers. The first author (AL) is a psychiatrist. The second (GB) has training in linguistics (including corpus linguistics methodology) and health care communication.

**Quantitative profile of the methamphetamine corpus.**

- Posts sampled (n=863)
- Total comments (n=8599)
- Total words (tokens) (n=370,452)
- Total unique words (types) (n=20,190)

### Ethical Considerations

All the data used in our analysis were posted in a public space, which are available to any internet user without having to subscribe or login to the site. The subreddit permits users to contribute anonymously with a pseudonymous username that is not linked to their offline identities. Our examination of the posts constitutes what Eysenbach and Till refer to as “passive analysis” [34]. The institutional review board at The Centre for Addiction and Mental Health (University of Toronto) reviewed the proposed study and opined that it did not require formal approval.

## Results

### Overview

As described in the previous section, our analysis began with the identification of keywords. Table 1 presents the keywords obtained from our corpus through comparison with EnTenTen20, which is manually grouped into thematic and lexical categories based on inspection of their uses in the contexts of the subreddit posts. All *P* values were <.001.

The most statistically marked keyword in the corpus is *meth*. It occurs 1826 times in the corpus with a relative frequency of 4923 per million tokens. The most frequent bigram containing *meth* is the copular “meth is” which occurs 99 times. Examination of concordance lines containing this bigram reveals considerable ambivalence among group contributors regarding the relative merits and hazards of methamphetamine. When describing its harms, contributors focus on the embodied harms of the substance. Note that examples were selected because they were deemed by the researchers to be representative of the patterns being described. To retain their integrity, extracts are reproduced with the contributors’ original spelling and punctuation.

*That’s how fucked **meth** is. We are it’s victim for life.*

*Even pharmaceutical** meth** is bad for you, the shit we use on the street is pretty much poisonous*

*I would stop snorting and hot railing for a while. Your nose is very irritated, and **meth** is worse for your nose than most drugs. Pretty caustic*

*In my opinion/experience, **meth** is more addictive and with higher abuse potential than adderall dexedrine, etc, not because it produces highs adderall cannot, but bc 1) its cheaper and easier to get, so you'll have a larger stash, and less incentive to not waste it since you won't be forced to wait till end of month to re up. 2) you can smoke it.*

In other instances, contributors use the “meth is” bigram to highlight the corporeal pleasures of methamphetamine use.

....**meth** is fun and leads to good sex and lots of energy is all I know

There have been studies that found as long as you don't let it fuck with sleeping, eating and taking care of yourself in general, **meth** is no worse for you than caffeine is in adults with no predisposed health issues.

I definitely wouldn't say anyone SHOULD do meth, but from what I know about coke - which admittedly, I haven't done coke - **meth** is a lot more sustainable.

*Meth* is by far the most common lexicalization used by contributors to describe methamphetamine but a richly textured lexicon permits nuanced descriptions of substances. Although synonymous with meth, the longer and more scientific-sounding synonym *methamphetamine* is sometimes invoked by exponents of a more formal, neuroscientific kind of discourse.

Probably because **methamphetamine** does not cause hallucinations. They come from lack of sleep or from stimulant psychosis not from the pharmacokinetics of **methamphetamine**.

I know that they sometimes actually literally will prescribe **methamphetamine** HCl in pill form for certain ADHD patients, that is, Desoxyn in two 20-25mg spaced doses.

Even less frequently, contributors use *desoxyn* as a synonym for *methamphetamine*. Desoxyn is a brand name for pharmaceutical methamphetamine. Almost all instances of this lexicalization of the substance refer to pharmaceutical and other medical uses, frequently in the context of treatment for attention-deficit/hyperactivity disorder (ADHD).

ADHD is treated with desoxyn in like 5 mg - 40 mg doses or some shit like that, once per day.

Went to docs and got diagnosed lol Adderall is good but **desoxyn** is the shit! Unfortunately its not easy to get.

The 3 keywords *meth*, *methamphetamine*, and *desoxyn* refer to the same chemical compound. However, these synonyms allow contributors to invoke or obscure a neurochemical discourse and a more or less formal register. Although the material referent is identical, the sense of each lexicalization is distinct. These divergent forms of expression permit nuanced relations between the material elements of the assemblage in question.

The use of different lexical types to describe methamphetamine highlights the different senses. As Keane notes, “In Plato’s Pharmacy Derrida highlights the undecidability and ambiguity of the Greek word pharmakon, which can mean both poison and cure” [32]. The tension evoked by the same compound functioning as both a toxin and a therapeutic or pleasure-causing agent is managed by using different lexical types, each of which exhibits a distinct discourse prosody.

Finally, the keyword *dope* is also used to refer to methamphetamine in the study corpus. However, of all lexicalizations of methamphetamine, *dope* is most highly polysemous, contested, and at times, ambiguous.

In a thread where a contributor refers to methamphetamine as *dope*, an interlocutor responds: “meth ≠ dope. dope is heroin or other strong downers.”

To this, another poster replies:

What region? Here in the Midwest, meth is absolutely 100% **dope**. **Dope** is definitely not heroin or any downers. **Dope** = shit = crystal, at least here. And that's the standard In every circle I've been involved with - old heads, heroin addicts, tweakers, teachers, shooters, smokers (although people who only fuck w weed/occasionally psychedelics like to call weed **dope**, and if pressed, will usually agree with you. But that's because they literally have no idea, tho)

Depending, then, on the context of the interaction, *dope* can refer to methamphetamine, but it can also refer to marijuana or to cocaine, heroin, or other opioids. In some instances, *dope* does not refer to a substance at all, but to a positive quality as in:

That color is so dope. Nice.

They look dope, very nice lamp-work

*Adderall* also appears as a keyword and occurs 90 times in the corpus. Like desoxyn, *Adderall* is a commercial trade name and refers to a preparation of dextroamphetamine and racemic amphetamine. It is used therapeutically in the treatment of ADHD and narcolepsy. Table 2 shows the collocates of this keyword.

Collocation analysis of *Adderall* reveals that *meth* is its only lexical (ie, nongrammatical) collocate. This pairing occurs because contributors compare *Adderall* and *meth:*

Dude, I flat out have conversations with voices, it’s like my favorite musicians and shit telepathically speaking to me, I enjoy to hear them, and meth and **adderall** all are similar but I feel way more euphoric on meth.

**Adderall** where I was, was going for fucking $10 for the 30mg shit. A gram of good meth is $40. Met someone who noticed I took the addies recreationally as well to study. Asked. Told me that **Adderall** is just smart meth.

**Table 1:** Keywords grouped into thematic or lexical categories and ranked by keyness score.

Thematic or lexical category	Associated keywords (raw frequency, keyness score)
Substances and chemicals	*meth* (1826, 1802); *adderall* (90, 179); *dope* (211, 121); *benzo* (51, 116); *amphetamine* (65, 100); *fent* (37, 99); *dopamine* (96, 87); *stim* (38, 80); *opiate* (66, 75); *methamphetamine* (65, 73); *n-iso* (24, 66); *fentanyl* (48, 65); *coke* (118, 58); *desoxyn* (21, 57); *Seroquel* (20, 50); *stimulant* (49, 50)
Psychological states	*psychosis* (150, 150); *comedown* (46, 117); *sober* (159, 89); *euphoria* (40, 50)
Descriptions of consumers of methamphetamine	*tweaker* (118, 288); *addict* (213, 63)
Mode/implements of administration	*roa* (68, 141); *snort* (178, 136); *bong* (78, 118); *boof* (36, 94); *crackback* (34, 92); *shard* (102, 87); *boofing* (29, 79); *tho* (141, 78); *redose* (20, 55); *hotrail* (19, 52); *vape* (35, 52); *smoke* (675, 52)
Internet-related lexis	*lmao* (137, 230); *idk* (108, 156); *subreddit* (42, 86); *lol* (630, 84); *Reddit* (84, 79); *cuz* (76, 59), *tbh* (46, 69); *lmfao* (25, 61); *wtf* (57, 53); *ur* (109, 52); *haha* (99, 50)
Swear words	*shit* (952, 146); *fuck* (975, 88); *fuckin* (66, 86)
Forms of address	*bro* (168, 107); *homie* (44, 89); *bruh* (29, 75)

**Table 2:** Collocates of *Adderall* ranked by cubed mutual information.

Rank	Collocate	Collocation frequency	Cubed mutual information
1	*and*	27	13.04
2	*was*	11	11.53
3	*meth*	11	11.30
4	*would*	7	10.68
5	*for*	11	10.54

### Affective Assemblages

As noted by Duff [22], “[a]ll drug assemblages should be regarded as affective entities in as much as affective processes are at least partially responsible for the formation of the assemblage.” Used in this sense, *affect* refers to emotional states and also refers to a body’s capacity to act upon or be acted upon, by other bodies. Our keyword analysis identified 4 lexical types which, when analyzed in their wider contexts of use, seemed to correspond to this broad definition of affect: *psychosis*, *comedown*, *euphoria,* and *sober*.

### Psychosis

Table 3 shows the collocates of the first of the affect keywords, *psychosis*.

Collocation analysis of *psychosis* reveals that *induced* is its most frequent collocate, typically occurring in the L1 position (ie, immediately preceding the node) to form the bigram induced *psychosis*. The term *drug-induced psychosis* is a clausal nominalization in that it converts a process into a noun and identifies a drug as the cause of psychosis. This diagnostic category and attendant causal explanation are consistent with both the Diagnostic and Statistical Manual of Mental Disorders, Fifth Edition, Text Revision (DSM-5-TR) and International Classification of Diseases 11th Revision (ICD-11) diagnostic classification systems. The function of this is to reproduce, within this context, the way in which methamphetamine and addiction are discursively represented in some research and policy practices [33].

Finally found medications and treatment that worked and officially diagnosed schizophrenic, since i was sober when the long lasting fully documented episode happend it wasnt considered drug induced psychosis.

Recent editions of the DSM have strived to be atheoretical [35] and to engage in “pure” description without invoking etiological explanations. However, attempts at description necessarily invoke assumptions regarding pathology whether implicit or explicit. In the case of substance-induced psychosis, the etiological formulation is included in the name. The use of the term *drug-induced psychosis* enacts the collateral reality that drugs cause psychosis.

By using this terminology, this excerpt provides an example of cases in which users enact the collateral reality that methamphetamine use causes psychosis. At the same time, it challenges that reality by alluding to the performative function of psychiatric diagnosis by using the modalizing mental verb *considered*. In so doing, this post challenges the reification of the diagnostic category and acknowledges that diagnoses are at least partially made in practice.

The causes of psychosis are also contested in the corpus. A medicalizing discourse of psychosis views it as a simple cause and effect, whereby methamphetamine use inevitably leads to psychosis. In contrast, contributors view the development of psychosis as resulting from a more complex assemblage that includes excessive methamphetamine use along with other habits and practices.

I used to get way too high and around day 3 of being up and overamped, I'd sit there and be unable to move. It's just a matter of moderation and how many days somebody is up. I eventually went into a bad **psychosis** from not staying hydrated, not eating, not sleeping, and not moving. Went deep into my head. But tonight for example I'm moderating what I smoke and I've done all my laundry and cleaned my house. For me, less is more.

I'm off the shit finally, but I know that **psychosis** is caused by nutrition and rest deficiency.

I think the longest run I've done is 11 days without sleep; but I was able to fend off **psychosis** by eating three meals a day and shutting down for 4-5 hours a day where it's laying in bed with low stimuli, so even if I'm not sleeping, I'm resting my spine, joints, and mind.

As said a million times over, the best way to fend off **psychosis** is regular sleep and healthy meals.

These posts present psychosis as the result of a complex array of corporeal factors, such as hydration, food, sleep, and activity level. They formulate psychosis as part of an assemblage that narrows and limits the range of activities that a body can do. Descriptions of positive experiences of methamphetamine use endorse an expansion of capacity. However constructions of experiences and activities situate a “bad psychosis” as being part of a diminished capacity to act. Contributors also use the verbal construction *fend off* to invoke the metaphor of battling an assailant. Metaphor is pervasive in the language used to reify mental states and forms of distress [36].

The notion that sleep deprivation is a necessary condition for methamphetamine use to cause psychosis is highly prevalent in our corpus. This belief is directly at odds with generally accepted medical knowledge, and a recently published scholarly review of methamphetamine-induced psychosis unequivocally stated that “methamphetamine and amphetamine can result in a paranoid psychosis…and the syndrome is not due to sleep deprivation” [37].

Although most descriptions of psychosis are negative, a few are positive:

That is cool psychosis and a lot of people don't say it if you get sleep I'm like you I like day three and four the best if you learne to handle it you enjoy the **psychosis** it's fun for me anyway the whole place turns into what I call non-comporeal beings.

When I started using Meth, I enjoyed the **psychosis**.

**Table 3:** Collocates of *psychosis*, ranked by cubed mutual information.

Rank	Collocate	Collocation frequency	Cubed mutual information
1	*induced*	6	14.55
2	*the*	43	14.21
3	*experiencing*	5	13.99
4	*and*	25	12.02
5	*from*	12	11.81

### Comedown

Contributors use the term *comedown* to refer to the affective and corporeal experiences that follow a period of methamphetamine use. In describing the nature of the methamphetamine comedown, contributors refer to other substances as a point of comparison.

**COMEDOWN**: first let's state that coke made me feel empty and anxious, molly made me feel depressed and meth makes me feel very on edge and paranoid and very nervous.

You get this weird gut anxiety which is different compared to other comedowns and it can be off setting but what goes up just comedown especially at this level.

Contributors discuss various strategies to mitigate the intensity of the comedown. Some contributors recommend vitamins, energy drinks, or nutritional supplements.

In future, oral dosing lasts a lot longer, the **comedown** is a lot smoother, and it's a lot less obsessive (you just eat 20mg instead of spending hours upon hours chasing the rush).

You need some **comedown** candy's and to accept that fact that. 1. Sleep is really the only thing that will help you 2. You're going down for a day or two.

I'm fucking spun, I grabbed some Tina, some raw coke, and a Xan for the **comedown**.

I'm interested in knowing since as ssri it makes serotonin relapse slower, may it increase the high or make the **comedown** easier when taken single dose after meth in not that high doses?

While accounts of methamphetamine addiction enacted in research and policy emphasize risks and risk mitigation, consumer accounts emphasize the role of pleasure in narratives of psychological addiction to methamphetamine [38]. Contributors used the keyword *euphoria* to describe the intense pleasure associated with methamphetamine ingestion. In response to a thread asking: “Should I sleep or load one more bowl 
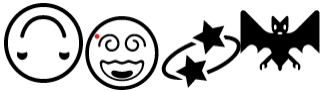
.”

One contributor responds:

Man if you've been up for more than a day or two already it should always be sleep. Redosing isn't even fun at that point, just keeps you awake and any feelings of **euphoria** have gone to bed.

This response navigates the tension between binging on methamphetamine, which lies outside the responsible ideals of normal life and the more responsibilized agency and subjectivity of such normative temporal routines as sleep. Contributors recruit discourses of neuroscience invoking the action of neurotransmitters to account for *euphoria* of the lack thereof,

You don't feel any **euphoria** and only feel tweaky because all the dopamine has been converted into adrenaline.”

Another contributor invokes a neuroscientific discourse to account for the euphoric effects of methamphetamine:

But initially, and if taking a healthy dose, its similar to most add meds just with a longer half life. Also, meth penetrates the BBB way faster and harder so you get the added euphoria.

By invoking the term *half life*, borrowed from pharmacology, and a reference to the blood–brain barrier, this post invokes a neuroscientific discourse in explaining the euphoric effect of methamphetamine use. As previously noted [33], consumer accounts of methamphetamine use enact several collateral realities, one of which is that methamphetamine use affects the brain. It also positions the contributor as belonging to a community of knowledge that includes consumers as well as scientists [33].

There is **euphoria** but it's not the main aspect of it all to me. More than anything, meth feels like I want to keep doing whatever it is I am currently doing for the whole day.

This post highlights that the motivation for methamphetamine use extends beyond an experience of pleasure as a benefit. It apprehends the “dynamics of space, embodiment and practice” [39]. The post speaks to the pleasure of practice or activities enabled by methamphetamine use. Although methamphetamine use is characterized by a sought-after, intensely pleasurable corporeal state, in order to construct the assemblage of methamphetamine use, it must occur in concert with practice.

Several of the keywords including *snort, boof, hotrail, vape,* and *smoke* refer to different modes of administering methamphetamine. *Snort* refers to nasal insufflation of methamphetamine crystal. *Boof* refers to rectal administration. *Hotrail* refers to nasal inhalation of methamphetamine smoke. To *vape* refers to the inhalation of a vapor formed by heating methamphetamine to the point that it evaporates but not so hot that it burns. Finally, *smoking* refers to inhaling smoke formed by the combustion of methamphetamine.

*Redose* refers to administering methamphetamine sometime, typically several hours, after a previous dose, when the effects of the earlier dose begin to wear off.

I am losing control. I compulsively **redose** and neglect important human needs such as hydration, hygiene, nourishment, among other things.

I usually just IV now. It goes from pure excitement to HOLY FUCK INTENSE as I shoot up, and it stays intense or an hour or two, tapers off for a good while and then I’m straight. I do have the urge to **redose** after the intense part wears off.

Beware though, the transformation into a mechanized tweakazoid **redose** robot isn't limited to the ritual of freebasing.

The implication that *redose* involves the repetitive ingestion of methamphetamine hints at the habitual pattern of some methamphetamine use. These posts suggest that using habitually opens the door to a pattern of use where using overshadows other social and occupational activities, leading to a problematic pattern of use.

Contributors associate the corporeal practices associated with different routes of administration with variations in the experience of intoxication. One post reads:

*I am gonna stop smoking this shit.*

It’s such a waste and it’s very fiendish. I can easily smoke 2 grams in 1 night. Plus I am waay to obsessed with forming a perfect crack back. It fucks with my head tbh.

A response to the thread offers:

Yup. It turns you into a re-dosing robot, long after the **euphoria** is gone. Don’t be a slave to the drug. Consider taking it orally in one dose instead.

The suggestion connects the corporeal practice of administration with the affective experience of intoxication.

### Lexicalizing Methamphetamine Users

Contributors use the term *addict* and *tweaker* to refer to people who habitually use methamphetamine.

The most statistically marked collocate of an *addict* is *functional*, occurring almost exclusively in the L1 position to form the bigram, *functional addict* (see Table 4).

First thing you gotta do is get a morning routine. It’s possible to be a **functional addict** you just gotta force yourself to take breaks so you can realize things like this.

Sure it's possible to be a **functional addict**, or even an occasional user.

Here again, contributors emphasize that stabilizing routines tend to promote an assemblage of less destructive use.

Tweaker is used to refer to people who habitually use methamphetamine. It refers to a more stigmatized subject than an addict. Posts referencing tweakers equate this identity with mental illness:

I had a **tweaker** girl tell me some wild story about her first hand experience with the fent cut meth, but she seemed like a nutball so I took what she said with a grain of salt at the time.

thank you. i didn't make it though, my friend who is like the most retarded, mentally ill, **tweaker** out there somehow made this thing. if i ever get around to making my own (which i probably should bc i dont trust anything that comes from him) then i'll use that tip ;)

Ya paranoid **tweaker** fuck.

Posts referencing tweaker also focus on the embodied stigmata of chronic methamphetamine use including psychomotor agitation, grooming, and skin lesions:

Just don't look like a **tweaker** and you're set.

If you've ever met a **tweaker** who sleeps you'll see that they're actually not some twitchy dude looking over his shoulders at 1,000Mph itching his friends head because his back has bugs coming out of his skin.

That's what separates a thriving user from a **tweaker** or junkie, self preservation and self respect.

**Table 4:** Collocates of *addict* ranked by cubed mutual information.

Rank	Collocate	Collocation frequency	Cubed mutual information
1	*functional*	12	17.26
2	*meth*	16	12.80
3	*drug*	8	12.05
4	*full*	5	11.99
5	*and*	18	11.16

## Discussion

### Principal Findings

The discursive patterns identified in this corpus of interactions problematize several of the collateral realities enacted by pre-existing scholarly research on methamphetamine use. As noted by Dwyer and Moore [40], extant drug education and research texts adopt a “pharmacology as destiny” approach to the relationship between methamphetamine and psychosis. This approach privileges pharmacology as the key determinant of experienced effects and discounts the importance of set, setting [41], and other contextual factors in drug experiences generally and psychosis in particular. It foregrounds a Humean constant conjunction [42] between methamphetamine use and psychosis. These discursive practices construct conventional ontological understandings of methamphetamine as anterior, singular, stable, and definite, and of its psychological effects as indicative of pathology. In contrast, the first-hand descriptions of psychosis provided by contributors conceptualize psychosis as existing as part of a complex assemblage. They posit that when methamphetamine use occurs in a complex of behaviors that includes regular sleep, healthy nutrition, meaningful activities, and a moderate degree of stimulation, then it does not necessarily include psychosis. This perspective is at odds with that of the existing scholarly literature on amphetamine-induced psychosis [43]. This insight may be helpful in guiding harm reduction efforts relating to methamphetamine psychosis by directing attention to the activities that occur alongside methamphetamine use, rather than solely toward the quantity used or the route of administration. This also has important implications for future research which may attempt to disentangle the complex relationship between methamphetamine use, psychosis, and the array of other factors that bear upon them.

The reasons for this apparent conflict in perspectives are not clear and merit further investigation. Studies concluding that amphetamines cause psychosis were conducted in laboratory settings with controlled conditions. In the real world, methamphetamine use is a complex, multifaceted, and changing phenomenon. As suggested by Law [44], experimental methods are ill-suited to address things that are emotional, ephemeral, elusive, or indistinct. We submit that real-world drug use, intoxication, and addiction are messy phenomena that defy clear and definite characterization or explanation.

This study found that methamphetamine users can choose from a rich and varied lexicon to refer to methamphetamine and other substances. In particular, lexical choices highlight that amphetamines can be seen as a life-giving elixir, an invigorating tonic, a prescription medication sanctioned by medical authorities, or a poison that harms and robs those who use it of agency. Contributors perform these different amphetamine objects by using different lexical items and by situating them in purposive discursive contexts. These include informal terms such as *meth* and *dope*, as well as more formal terms including *methamphetamine*. Use of the lexical item *dope* can be used to signal “in group” status in the community. Users also invoked terms derived from the commercial production, marketing, and sale of stimulants. In doing so, they frequently invoked a more formal neuroscientific register and positioned themselves as members of a community of knowledge that includes scientists as well as drug consumers. This may be a feature of the kind of web-based context represented in the corpus data assembled for this study since such environments have provided the contextual basis for observations of the discursive enactment of so-called “expert patient” identities in previous studies of digital health-related communication [25,45,46].

Contributors described affective states associated with the use and the cessation of methamphetamine use. Affective states such as euphoria represent the bliss of amphetamine ingestion, while comedown represents its opposite. Contributors construed these two affective states as existing in tension, each opposite being a necessary counterpoint. Psychosis was generally framed as undesirable and contributors traded in strategies to avoid psychosis. However, a minority countervailing viewpoint challenged that characterization and identified that psychosis can be an exciting novel experience.

Contributors also use lexical choices to construct symbolic boundaries [47] between various types of methamphetamine users. As noted by Copes et al [48], people who use methamphetamine are highly stigmatized and make distinctions between different types of users based on their ability to maintain their physical appearance, mental health, and daily obligations. The use of terms such as *functional* addict is presented as desirable in contrast to *tweakers* who are presented in stigmatized terms as having lost control of their use. This echoes previous findings that individuals negotiate the boundaries of acceptable drug use by aligning their identity with the virtues of autonomy, control, and responsibility and distancing themselves from such stigmatized subject positions as addict or junkie [2].

### Limitations

A limitation of our analysis is that we have only studied 1 subreddit. Future research should consider a wider range of interactional contexts—including other sites and sites using languages other than English—in order to ascertain the extent to which the patterns observed in this study might be a feature of the culture and subculture (ie, this particular subreddit) we studied, and the extent to which they might be considered part of a broader discourse around methamphetamine use. Furthermore, it is possible that other subreddits on meth use exist on topics that may have different positive and negative sentiments about use and behavioral-related topics or may have different types of contributing users. This brings us to another limitation, while the anonymity of the site we studied was, on one hand, an advantage for the purposes of our analysis (potentially giving rise to more candid disclosures, as discussed earlier), a limitation of such anonymity is that it is not possible for us to reliably assess the sociodemographic make-up of the users represented by our data, nor to draw comparisons between groups. Addressing these limitations would require to use of different—possibly even purposively designed—data sets. It is our intention that the findings reported in this paper will provide a springboard for such work and a wider program of research to better understand the discursive dynamics of addictive substance use.

### Conclusions

In summary, this study analyzed digital discussions among methamphetamine users, which finds that the contributions making up the subreddit under study frequently challenged a preexisting research view that links methamphetamine use directly with psychosis. The contributors whose discourse we studied presented a complex array of factors including regular sleep, nutrition, meaningful activities, and stimulation, which, when combined with methamphetamine use, may not necessarily lead to psychosis. This “set and setting” perspective offers a fresh viewpoint on drug-induced psychosis and can guide future harm reduction strategies and research. Many previous studies might thus be viewed as overlooking the real-world complexities of methamphetamine use, perhaps due to the use of controlled experimental settings. Actual drug use, intoxication, and addiction are complex, multifaceted, and elusive phenomena that defy straightforward characterization. Notably, we found evidence of lexical choices whose use indicated a delineation of symbolic boundaries between types of methamphetamine users, establishing a societal hierarchy within the user community.

## Abbreviations

ADHD: attention-deficit/hyperactivity disorder
DSM-5-TR: Diagnostic and Statistical Manual of Mental Disorders, Fifth Edition, Text Revision
ICD-11: International Classification of Diseases 11th Revision
